# Jurassic surgery and immunity enhancement by alkyglycerols of shark liver oil

**DOI:** 10.1186/1476-511X-13-178

**Published:** 2014-11-27

**Authors:** Beniamino Palmieri, Alfonso Pennelli, Alessandro Di Cerbo

**Affiliations:** Azienda Ospedaliero-Universitaria Policlinico di Modena, Surgery and Surgical Specialties department, University of Modena and Reggio Emilia, via del Pozzo 71, Modena, 41124 Italy; Department of Biomedical Sciences, Faculty of Medicine G. d’Annunzio University, Chieti-Pescara, Italy; Poliambulatorio del Secondo Parere, viale Reiter 14, Modena, 41125 Italy; Scuola di Specializzazione in Biochimica Clinica, G. d’Annunzio University, Chieti-Pescara, Italy

**Keywords:** Alkylglycerols, Nutraceutical product, Immune reactivity modulation

## Abstract

**Background:**

Shark liver oil (SLO) contains both alkylglycerols (AKG) and squalene and is an ancient remedy among the fishermen on the west coast of Norway and Sweden. Literature reports showed that alkyglycerols enhance Fc–receptor mediated phagocytosis, increase humoral immune response and delay hypersensitivity reactions.

**Methods:**

On this background we performed an open spontaneous study on 40 very old aged surgical patients preoperatively treated with alkyglycerols (500 mg twice a day for 4 weeks), in order to reduce the risks of operation, counteracting the postoperative inflammatory and anergic conditions thus achieving quick and plain recovery. To better understand the possible therapeutic impact of alkyglycerols we compared on a case/control basis treated versus untreated patients submitted contemporarily to the identical operation and exposed to the same environmental and seasonal risks.

**Results:**

The onset of complications was reduced in the alkyglycerols treated group and the compliance to the natural treatment was excellent without any serious adverse effect. WBC count and IgG significant increase (respectively p <0.05 and p <0.001) might explain some sort of protection against infectious agents and wound repair adverse events. Also lymphocytes concentration significantly increased in the AKG treated group (p <0.001) whereas a slight decrease was observed in the control group. Conversely neutrophils significantly decreased in the AKG treated group (p <0.001) meaning that patients have no more infections and have re-established their physiologic state. However a significant increase was observed in the control group (p <0.05). CRP significantly decreased in the group receiving AKG (p <0.05), thus evidencing a slight antiinflammtory effect of the product. Also ESR decreased from a baseline in the group receiving AKG.

**Conclusions:**

In conclusion we suggest the opportunity to introduce this nutraceutical product in dosages of 500 mg twice a day to very old people before surgical treatment for an effective modulation of leukocytes and soluble immune reactivity according with the shark liver oil consumption trend in the northern Europe countries folk medicine. For this reason it might be advisable a wider study on a substantially bigger patients cohort focused on the complication rate prevention or control.

## Background

**Figure 1:**
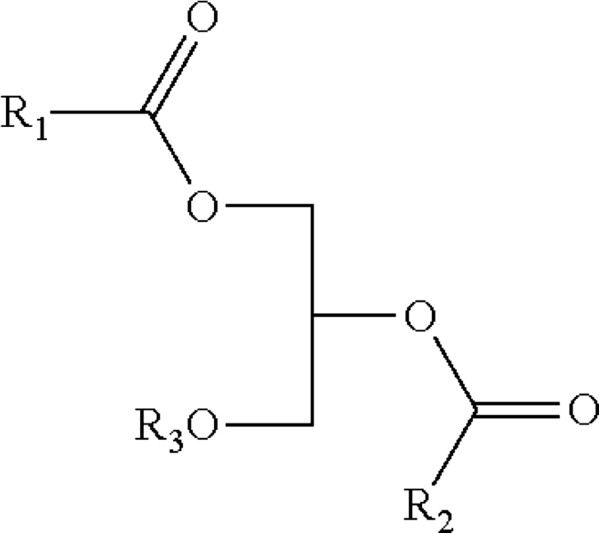
**AKG chemical structure.**

Shark liver oil (SLO) contains both alkylglycerols (AKG) and squalene and is an ancient remedy among the fishermen along the west coast of Norway and Sweden. It has been successfully used for wound healing [1], inflammatory lung [1] and alimentary tract [2] diseases, lymphadenopathy [3], cancer [4] and dermatitis [5]. In 1922 Tsujimoto and Toyama found AKG in SLO [6] and Sir Robert Robinson, a Nobel laureate, first synthesized them in 1930 [7]. In the natural sources, they are always found esterified with fatty acids. Structurally they are alkyl ethers of glycerol (Figure 1).

Brohult and Holmberg [8], using the unsaponifiable portion of different bone marrow fats as well as preparations containing esters of AKG in child leukemia, observed a pro-differentiating effect on the white blood cells leading to AKG clinical administration in actinic leucopenia [9]. In the early 1950s Astrid Brohult began experimenting with AKG from the calf marrow on children with leukaemia, highlighting their property to stimulate white blood cells production and later, in 1963, she published a thesis on AKG and their use in uterine cancer irradiated patients demonstrating that AKG administration during radioactivity exposure reduced the bone marrow induced leukopenia following radiation therapy for uterine cervix carcinoma [10]. AKG treated patients showed a reduced complication rate and 47% fistula incidence reduction when AKG had been administered prior to radiation treatment [11, 12].

The principal AKG include chimyl (hexadecyl), batyl (octadecyl) and selachyl (octadecyl) ethers. Hallgreen et al. reported that glycerol ethers occur in the tissues in the form of diesters and alkyl acyl phosphatides [13]. 1-O-Alkylglycerols and 1-O-(2-methoxyalkyl) glycerols were isolated from the neutral lipids and phospholipids of human colostrum, human milk, cow’s milk, sheep’s milk, human red bone marrow, red cells, blood plasma, and a uterine carcinoma (Table 1).

**Table 1:** **AKG percentage in human bone marrow**, **human milk and liver oil**

Alkylglycerols	Human bone marrow	Human milk	Greenland shark liver oil
14:0			2.0
15^a^			0.7
16:0	29.4	23.9	9.1
16:1		Trace	10.8
17 ^a^	7.6	3.6	3.6
18:0	24.6	22.8	2.8
18:1	16.7	33.8	59.4
18:2		1.4	1.6
18:3			
19^a^	6.1	2.4	1.5
20:0	2.9	1.6	
20:1	3.2	2.3	6.2
22:0	0.7	0.7	
22:1	5.1	3.4	2.2
24		2.1	

The number of carbon atoms in the first column refers to the long-chain component of the molecule. The number after the column denotes the number of double bonds. ^a^Both branched and normal chains C_15_, C_17_, and C_19_ are present [1].

AKG and alkyl lysophospholipids significantly activate cytotoxic macrophages leading to enhanced Fc–receptor mediated phagocytosis and increase humoral immune response and delayed hypersensitivity reaction [14]. AKG have been shown to stimulate hematopoiesis, erythropoiesis, thrombocytosis and granulocytosis in experimental animals [15]. The anticancer effect of AKG might be due, according to the previously described studies, to the property of activating macrophages, and increasing the cytokines production such as Interleukine-12 (IL-12) and Interferon-gamma (IFN-γ). Recruitment and activation of macrophages, in fact, is acknowledged to be fundamental in the primary antitumor defence [16]; while IFN-γ, (a T-cell derived lymphokine), inhibits a wide number of malignant cells either directly counteracting cancer cells growth or through its immunomodulatory properties [17], IL-12 induces IFN-γ secretion from naive and activated T and natural killer (NK) cells, enhances the cytotoxic activity of NK cells, cytotoxic T lymphocytes and lymphokine activated killer cells, increases of the proliferation of pre-activated T cells and NK cells, with a key role in the tumors defence [18–20]. On this basis, considering the potential benefits that AKG administration might have on very old pericentenarian patients submitted to surgery (we enclosed this ultra aged cohort of surgical candidates in the naïve term of “Jurassic surgery”), we administered such compounds to 40 very old patients before surgical intervention in order to reduce the risks of operation and to achieve the best compliance and quick recovery.

We run an open spontaneous study on 40 operated cases comparing treated versus untreated patients undergone the same type of operation during the same historical period and the same environmental and seasonal risks.

## Results and discussion

**Figure 2:**
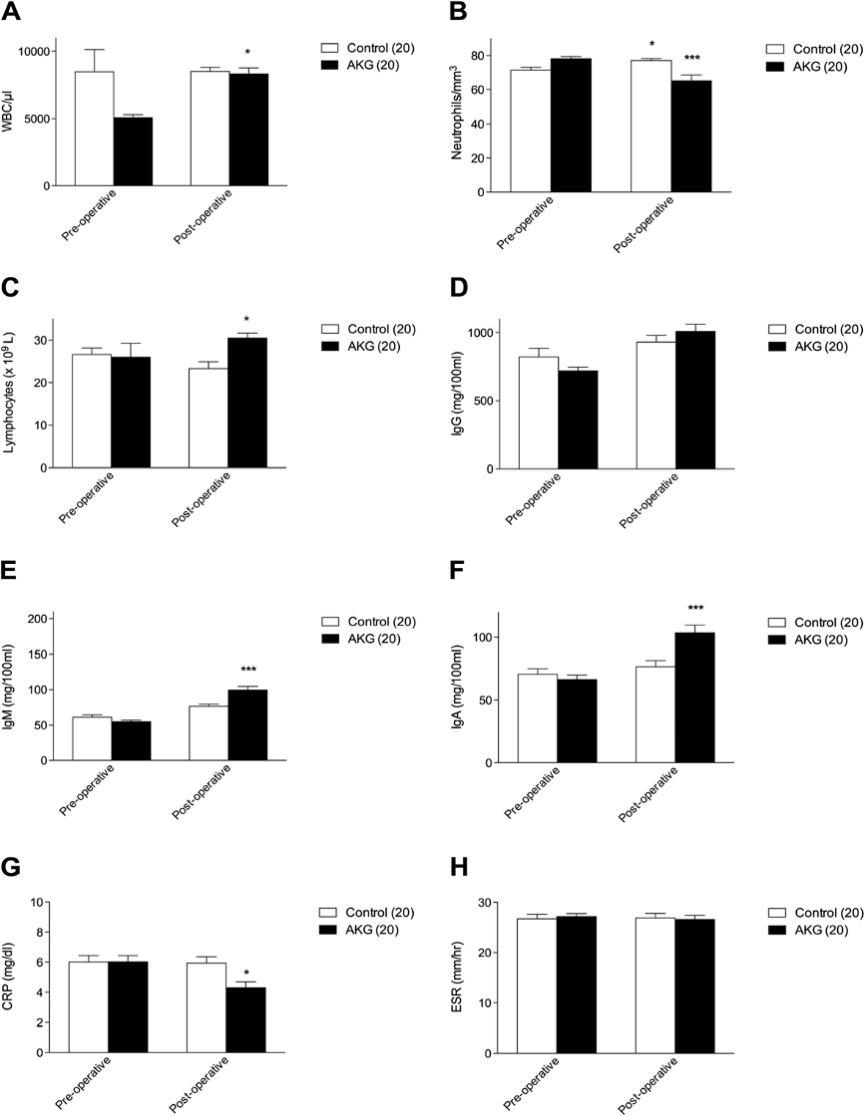
**Measurements of biochemical parameters. A**. Schematic representation of the improvement in leukocytes in the AKG treated group meaning a enhanced physiological response to the surgical trauma and wound remodeling; **B**. Schematic representation of the decrease of neutrophils in the AKG treated group meaning the absence of infections and a re-established physiologic state; **C**. Schematic representation of the increase of lymphocytes in the AKG treated group; **D-F**. Schematic representations of the improvement in IgG, IgM and IgA respectively in the AKG treated group, meaning an activation of plasma cells primed for specific antibody response and a definite reaction against sorting infectious agents, respectively; **G**. Schematic representation of the decrease of CRP in the AKG treated group meaning a slight antiinflammtory effect of the product; **H**. Schematic representation of the decrease of ESR in the AKG treated group *p **<** 0.05, ***p **<** 0.001.

Results clearly indicate a significant increase of WBC concentration was observed in the AKG treated group, from a baseline value of 5071 ± 236.4 per μl to 8309 ± 451.9 per μl (p <0.05), compared with the control as enhanced physiological response to the surgical trauma and wound remodeling (Figure 2A).

Post operative immunoglobulins dosage of AKG treated patients revealed significant increase of both IgM (Figure 2E), from a baseline value of 54.95 ± 2.26 mg/dl to 99.47 ± 5.31 mg/100 ml (p <0.001), and IgA (Figure 2F), from a baseline value of 66.4 ± 3.62 to 103.6 ± 6.18 mg/100 ml (p <0.001), as a definite reaction against sorting infectious agents either in the sutured wound area or in the respiratory and urinary tract (IgA). As to IgG an overall increase was observed both in AKG treated group, from a baseline value of 718.9 ± 27.34 to 1008.6 ± 52.69 mg/100 ml, meaning that the activation of plasma cells already primed for specific antibody response especially against infectious bacteria and fungi was occurred, and in the control group, from a baseline value of 822.2 ± 63.98 to 930.6 ± 49.17 mg/100 ml. Also lymphocytes concentration significantly increased in the AKG treated group from a baseline value of 26.0 ± 3.31 × 10^9^ L to 30.50 ± 1.13 × 10^9^ L (p <0.001); whereas a slight decrease, from a baseline value of 26.60 ± 1.60 × 10^9^ L to 23.30 ± 1.61 × 10^9^ L, was observed in the control group (Figure 2C). On the other hand neutrophils significantly decreased from a baseline value of 78.20 ± 1.19 mm^3^ to 65.20 ± 3.48 mm^3^ in the AKG treated group (p <0.001), meaning that patients have no more infections and have re-established their physiologic state. However a significant increase from a baseline value of 71.45 ± 1.64 mm^3^ to 77.15 ± 1.11 mm^3^ was observed in the control group (p <0.05) (Figure 2B).

CRP significantly decreased from a baseline value of 6.03 ± 0.41 mg/dl to 4.31 ± 0.38 mg/dl in the group receiving AKG (p <0.05), thus evidencing a slight antiinflammtory effect of the product (Figure 2G). ESR decreased from a baseline value of 27.18 ± 0.61 mm/hr to 26.60 ± 0.8 mm/hr in the group receiving AKG (Figure 2H).

The onset of complications such as broncopneumonia, pleuritis, thromboembolism and wound disruption was reduced in the AKG treated group and the compliance to the natural treatment was excellent without any adverse effect; moreover WBC count restoration and IgG increase might at least partially explain some sort of protection against infectious agents and wound repair adverse events.

In the control group, along with previously cited complications we also observed fever episodes almost due to local and systemic low level of both non specific and specific defence of these very old patients.

Very old aged surgical patients are quite fragile and require great care in order to avoid both localized or systemic infections. Based on the folk medicine experience, we tried the administration of AKG in the perioperative phase in order to evaluate if its well reknown immunomodulating activity, even in the extreme life decade, might be useful to prevent either local or systemic surgical complications. Our patients reacted positively to AKG administration and the compliance of the molecule was very high with no drop out due to the optimal tolerance and safety.

Notwithstanding this small cohort of aged patients, the concordant hemato-immunological effects of AKG administration reduced both the number and the severity of complications in the treated patients and was shown to be helpful for a quick and easy recovery.

## Conclusions

In conclusion we suggest the opportunity to introduce this nutraceutical product for an effective modulation of leukocytes and soluble immune reactivity according with the shark liver oil consumption trend in the northern Europe countries folk medicine. The shark liver oil, accordingly with the folk medicine tradition, administered in dosages of 500 mg twice a day to very old people before surgical treatment induced a significant increase of WBC, lymphocytes, IgM and IgA in the postoperative period. The compound had not any kind of intolerance or side effects, and the treated patients had a plain uneventful outcome notwithstanding the fragile condition due to the extreme life decade. For this reason it might be advisable a wider study on a substantially bigger patients cohort focused on the complication rate prevention or control.

## Methods

Forty patients (mean age ± SEM, 97.20 ± 0.86 years; 57.5% males, 42.5% females) were admitted to surgical outpatient office for a preliminary evaluation of intervention feasibility. They were affected by the following surgical diseases: umbilical hernia (2 cases), inguinal hernia (6 cases), lower leg varyces (4 cases), laparoscopic cholecystectomy (8 cases), breast cancer (2 cases), hydrocele (4 cases), phymosis (2 cases), skin cancer (2 spinocellular cancer, 2 basocellular cancer and 2 melanomas), laparocele (4 cases), giant lipoma (2 cases). Patients were equally distributed, in terms of affecting disease, in two groups, control (12 males and 8 females) and AKG (11 males and 9 females).

They were matched as case control along their recruitment period which lasted from January 2011 up to October 2013.

The preliminary selection of the patients to be treated was related to the finding of a low total WBC count, shift of the formula to neutrophilia (above 75% neutrophils) and serum immunoglobulin level. In order to prevent post op infection on a standardized basis we used ceftriaxone 1 gr pre op followed by the same dosage daily i. m. for the following 4 days. Antiinflammatory drugs were ruled out from the schedule and for the pain control we used only tramadol 100 mg 2-3 times a day and Phentanil (Durogesic 25, 50, 100 mg tailored on pain intensity), in order to avoid individual variations in biochemical parameters due to interfering molecules.

The investigated parameters, before and after 2 weeks of surgical intervention, were inflammation indexes (reactive C protein or CRP, erythrocyte sedimentation rate or ESR), total leucocyte number and formula and serum immunoglobulins (IgA and IgM).

The AKG (Lysi Italy, Manzano (UD), Italy; 500 mg of pure alkylglycerols per capsule) administration (500 mg twice a day) started at the first surgical admission visit and prolonged along 4 weeks, with temporary withdrawal from the morning of operation to the second post op days.

This study was performed in accordance with the Declaration of Helsinki and it was approved by the Institutional Review Board at the Poliambulatorio del Secondo Parere (Modena, Italy) were the study was performed.

### Statistical analysis

Data were analyzed using GraphPad Prism 6 software (GraphPad Software, Inc., La Jolla, CA, USA). All data are presented as the means ± standard error of the mean and were first checked for normality using the D’Agostino-Pearson normality test. A paired Student’s *t*-test was used to compare changes in values after 2 weeks treatment versus baseline. Difference in values between the two groups was analyzed using a two-way analysis of variance (ANOVA) followed by Sidak’s multiple comparisons test.

## Abbreviations

SLO: Shark liver oil
AKG: Alkylglycerols
NK: Natural killer
IL-12: Interleukine-12
IFN-γ: Interferon-gamma
WBC: White blood cells
IgM: Immunoglobulin M
IgA: Immunoglobulin A
CRP: Reactive C protein
ESR: Erythrocyte sedimentation rate.
